# Suicide, Stigma, and Utilizing Social Media Platforms to Gauge Public Perceptions

**DOI:** 10.3389/fpsyt.2019.00947

**Published:** 2020-01-13

**Authors:** Nila A. Nathan, Kalpana I. Nathan

**Affiliations:** ^1^ Independent Researcher, Mountain View, CA, United States; ^2^ Department of Psychiatry and Behavioral Sciences, Stanford University School of Medicine, Stanford, CA, United States; ^3^ Department of Psychiatry, Palo Alto Veterans Affairs Health Care System, Palo Alto, CA, United States

**Keywords:** suicide, stigma, social media, perceptions, attitudes, survey

## Abstract

**Introduction:** Suicide, a multifaceted complex outcome that arises from numerous biopsychosocial factors, is a public health concern which is growing in numbers despite valiant prevention efforts. There is still a lot of stigma surrounding suicide that needs to be addressed. Social media is growing exponentially and there are many forums where suicidality is being discussed. As a result, we conducted a brief survey on the perception of suicide on social media platforms of Facebook and Reddit in order to gather more information.

**Results:** Of the 152 respondents, 86% believed that suicide is preventable, and 72.85% believed that it is a person’s right to die by suicide. About a third (31.79%) had lost someone close to them to suicide. Respondents who did not think suicide was preventable also viewed suicide as either a sign of strength (42.86%) or a revenge act (33.3%). Those who responded that someone close to them died by suicide believed that the media glorified suicide (56.25%) while those who did not lose someone, did not believe that (66.99%). Women (61%) found social media to be a good platform for people to ask for help while men did not (60.61%).

**Conclusions:** We utilized the social media platforms to gauge the perception of suicide and found among the sample of mostly young white respondents, suicide is not stigmatized, most believed it is preventable and it is a person’s right to die by suicide. While women found social media to be a good platform to ask for support, men did not, which is in keeping with the trend that women tend to be more willing to seek help. A third of the group had lost someone close to them to suicide which was the national average, who tended to believe that media glorified suicide. Limitations of this study include the fact that those who respond voluntarily to a survey likely have an interest in the topic, and this might not accurately reflect the public opinion and attitude.

## Introduction

Suicide is a global phenomenon, with nearly 800,000 people dying each year and 79% occurring in low- and middle-income countries (1). In 2017, it is estimated that 47,173 Americans died by suicide, and around 1.4 million attempted suicide; men died by suicide 3.54 times more often than women and firearms accounted for 50.57% of all suicide deaths (2).

Society has viewed suicide through the lens of religion and politics, and this has historically undergone dramatic changes over time and has shifted public opinion in myriad ways. In the ancient Roman times, self-killing was recognized with the Epicureans acknowledging man’s fundamental right to dispose of his own life (3). In 1897, Durkheim published “Suicide: a study in Sociology” (4), which highlighted social conditions that were identified to be the root cause and shifted the focus away from suicide being considered an irredeemable sin or moral crime. Durkheim proposed four different types of suicide based on social integration and regulation: egoistic suicide, altruistic suicide, anomic suicide, and fatalistic suicide (5–7). In the past several decades, the pendulum has swung in the direction of a psychological model, with the assumption that nearly all suicides result from mental illness, although this is often diagnosed after the fact from psychological autopsies (8). Joiner’s interpersonal theory of suicide has received widespread recognition (9, 10), but has faced some criticism (11). Across different cultures, suicide has also been perceived to result from overwork or occupational burnout (12, 13), serious financial problems (14), lack of support (15), problems with mandatory military service (16), interpersonal relationship problems (17), and marital problems (18). Suicide has been associated with the stress of dealing with chronic medical conditions such as epilepsy and stroke (19, 20). It is helpful to gauge the attitude of the public towards suicide, its acceptance, condemnation, and preventability, and whether it can be encompassed in the argument for a person’s right to die.

Suicide has been steeped in stigma for centuries, and there are vestigial remnants of it to this day, mostly perceived by suicide loss survivors and suicide attempt survivors. Stigma is an overarching term that contains three elements: problems of knowledge (ignorance), attitudes (prejudice), and behavior (discrimination) (21). Public stigma is a social phenomenon that refers to the negative attitudes held by members of the public about people with devalued characteristics, while self-stigma is the internalization of these public attitudes which leads to numerous negative consequences (22). Stigma and discrimination in relation to diseases such as HIV can be measured through many dimensions such as blame/judgment (23), shame (24), discrimination in different settings, and disclosure of disease status (25). Common stereotypes about suicide are emotional weakness, attention-seeking, selfishness, malingering, and immorality. Those who attempt or die by suicide are perceived to be impious (not praying enough, believing enough), or as betraying family and others through cowardly or selfish acts (26). In addition to stigma, survivors’ guilt often compounds and reinforces the stigma either imagined or accurately perceived by bereaved friends, family, and therapists (27).

With the rapid technological advances, the use of social media platforms has grown exponentially, with Facebook, one of the platforms used for the study, having 2.41 billion active users per month (28). Social media platforms can offer several potential benefits in suicide prevention, which include reach, accessibility, nonjudgmental, and anonymous nature of such platforms. However, these platforms have also been used to spread information about how to die by suicide, help make cyber-suicide pacts with strangers, and access unregulated online pharmacies. There is the increased risk of media contagion effect, with peer pressure from online forums that are pro-suicide, swaying those who are ambivalent (29). Accessing and utilizing social media platforms can be a huge boon in prevention of suicide, but there are numerous ethical and methodological challenges surrounding privacy in the digital age for clinicians and researchers (30, 31).

Our study aims to understand the attitudes of social media platform users towards suicide, whether there are correlations with demographics and other factors such as having lost someone close to suicide, and whether these platforms for help.

## Materials and Methods

### Questionnaire Development

To better understand how the public views suicide in this current era, we developed a short survey to gauge the perception of suicide in social media. While at least 14 suicidal attitude scales have been identified, there is no gold standard due to the implicit instability of attitudes, the varied dimensions of attitudes, and a lack of consensus (32). The Attitudes Towards Suicide (ATTS) questionnaire has 34 items which include 10 dimensions (suicide as a right – justifiability, incomprehensibility, noncommunication, preventability, tabooing, normal-common, suicide as a process, relation caused, preparedness to prevent, and resignation) (32). The Suicide Opinion Questionnaire (SOQ) has 100 items assessing different domains, with a 5-factor structure: 1) Acceptability (AC): “People with incurable diseases should be allowed to commit suicide in a dignified manner.”; 2) Perceived Factual Knowledge (PFK): “Most suicides are triggered by arguments with a spouse.”, willingness to accept as fact inaccurate statements related to suicide; 3) Social Disintegration (SD): “The higher incidence of suicide is because of the lesser influence of religion.”; 4) Personal Defect (PD): “I would feel ashamed if a member of my family committed suicide.”; and 5) Emotional Perturbation (EP): “Most persons who attempt suicide are lonely or depressed.”, that those who attempt or die by suicide are emotionally distraught or mentally ill (33). The Cognition Concerning Suicide Scale (CCSS) has three factors, the first is “right to commit suicide” (CCSS-S 8 items: “Everyone has the right to commit suicide”, “When life consists of intolerable pain, suicide is an acceptable alternative”); the second factor is “interpersonal gesture” (CCSS-I 5 items: “I sometimes think suicide would be a good way to pay back people who have hurt me deeply”, “Taking my own life would be a good way to make sure I would always be remembered”); the third factor is “resiliency” (CCSS-R 4 items: “Even if I got tired of living, I would not seriously consider suicide as a way out”, “Even if I could not be with the person I love, I would not consider suicide”) (34).

The brief questionnaire we developed had items under these main themes: 1. Demographic characteristics (age, gender, ethnicity, level of education) 2. Had someone close to you died of suicide 3. Whether media glorifies suicide 4. Whether social media is viewed as a platform where people could get help and support 5. Whether suicide is preventable 6. Does one have the right to die by suicide 6. Attitudes about suicide that explore themes of acceptability, interpersonal gesture, stigma (it is a way to escape, a selfish act, a selfless act, an impulsive act, a sign of weakness of cowardice, a sign of strength or courage, a revenge act). In an effort to keep the survey short, we did not request open and qualitative comments (35).

### Administration

The survey was posted on social media forums of Facebook and Reddit, explaining the purpose of the survey and requesting voluntary and anonymous participation. There was no advertisement and no incentive for participation. Participants were advised to not take the survey if it in anyway was a trigger or caused discomfort. The survey included information about The National Suicide Prevention Lifeline which offers great resources, and can be reached by calling 1-800-273-TALK(8255) or chat online at https://suicidepreventionlifeline.org/. No personal data, such as name or other identifying information, were collected in the survey as a measure to protect personal information. The data was stored in a database accessed through surveymonkey, which was continuously updated during the survey period. Ethical approval was sought from the Institutional Review Board (IRB) of Stanford University School of Medicine, and all procedures were in compliance with the Helsinki Declaration. The first page of the survey asked for participants’ consent, and if the person consented, they were taken to a second page with 10 questions with the focus on perceptions regarding suicide. It was posted on social media for one week and we had a total of 152 respondents.

### Data Analysis

Descriptive statistics of the characteristics of respondents and responses to each question are presented. Analyses are based on the respondents’ age, ethnicity, level of education, and their perception of suicide. Normally distributed data were presented as a mean and 95% confidence interval (95% CI) or standard deviation (SD), whereas data that were not normally distributed were presented as a median and 95% CI. Analysis of Variance (ANOVA) was conducted separately to assess the individual associations of demographics, with attitudes towards suicide. Variables that were found to be significantly associated in the univariate ANOVA (p < 0.05) were highlighted.

## Results

**Figure 1:**
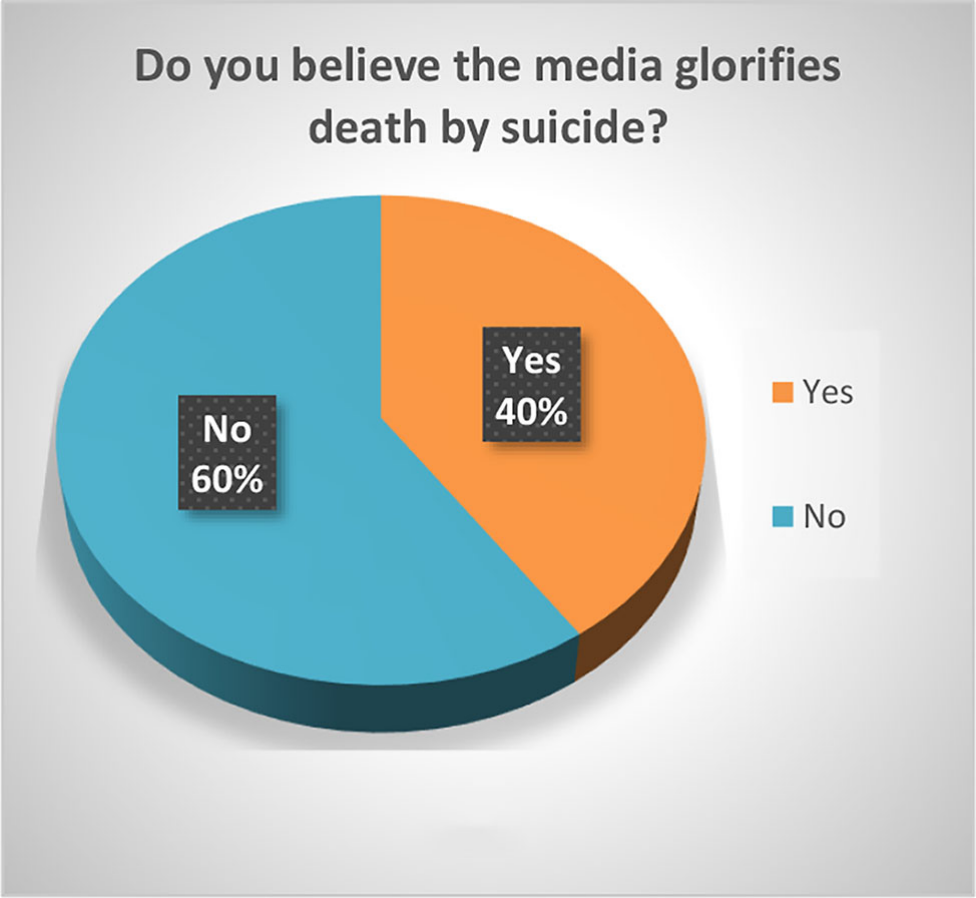
Do you believe the media glorifies death by suicide?

**Figure 2:**
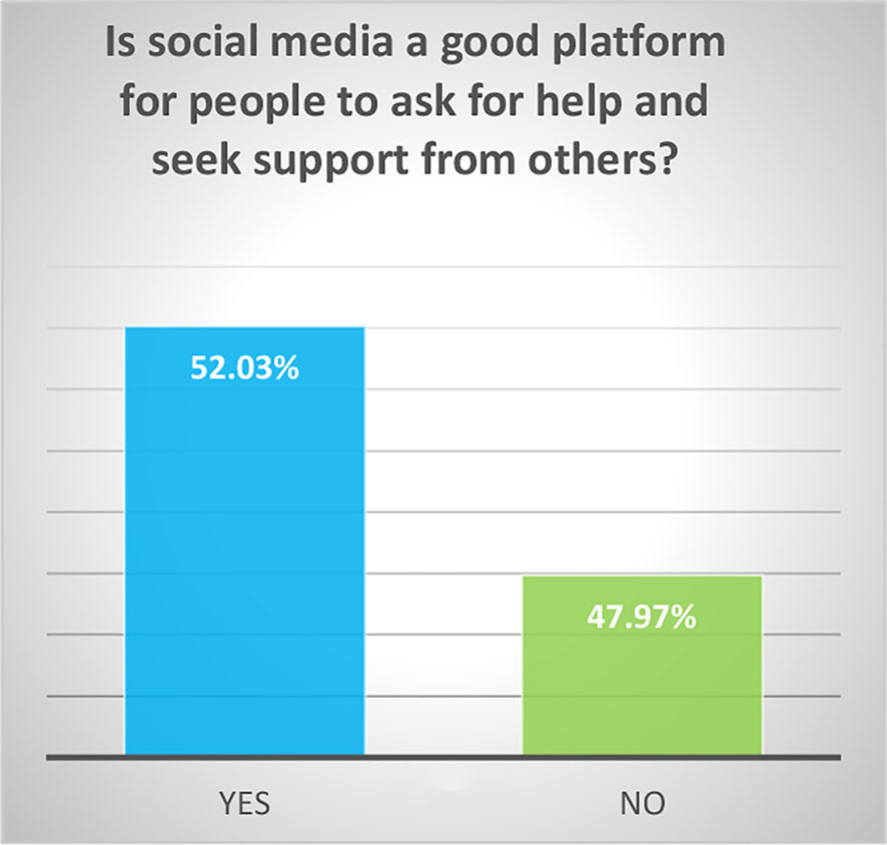
Is social media a good platform for people to ask for help and seek support from others?

**Figure 3:**
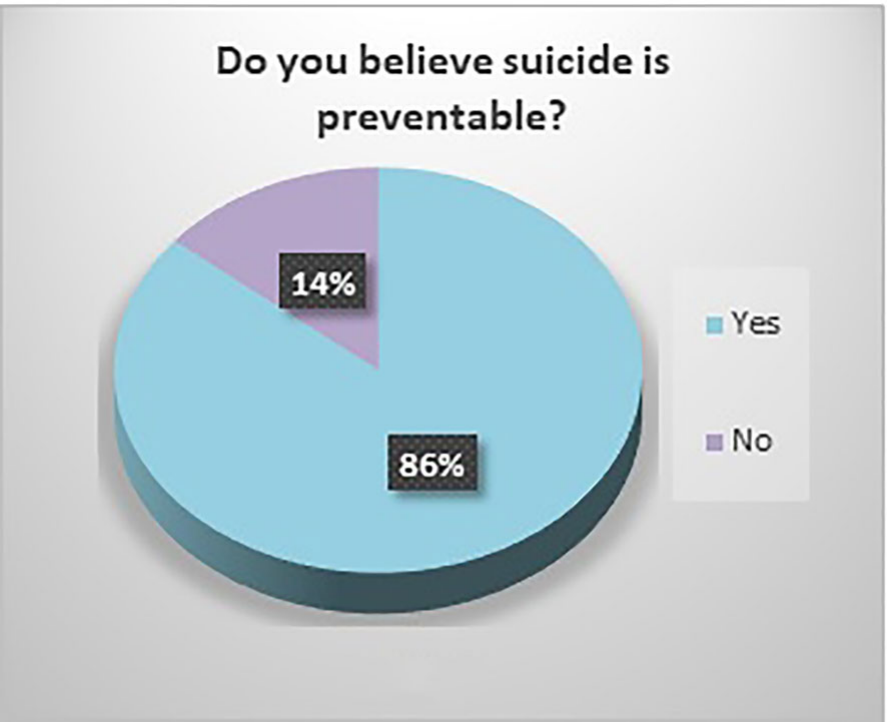
Do you believe suicide is preventable?

**Figure 4:**
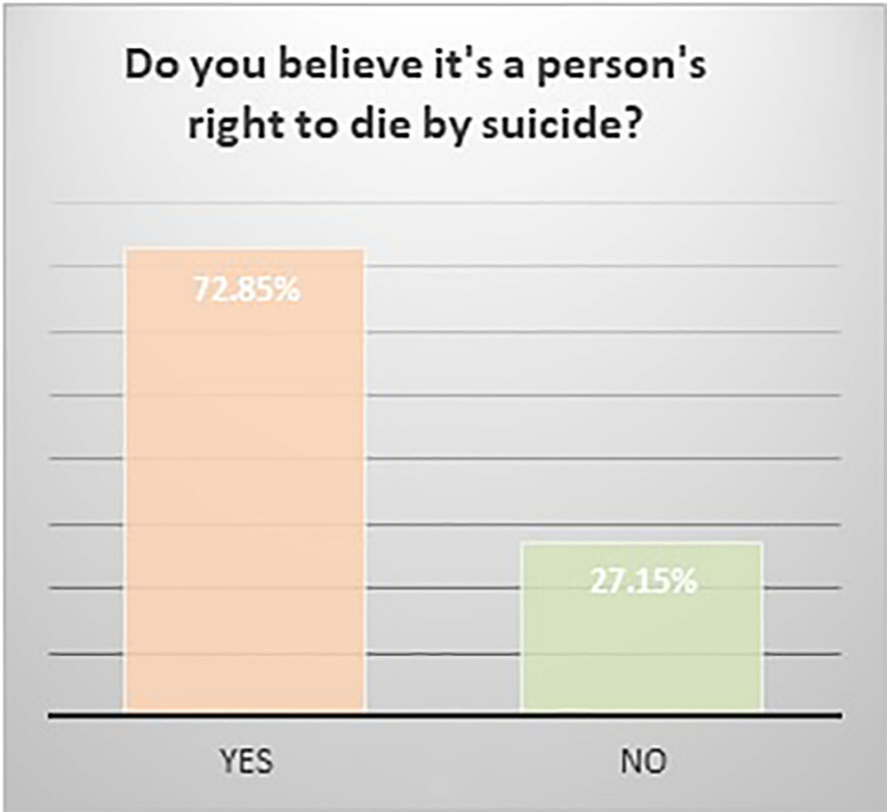
Do believe it's a person's right to die by suicide?

**Figure 5:**
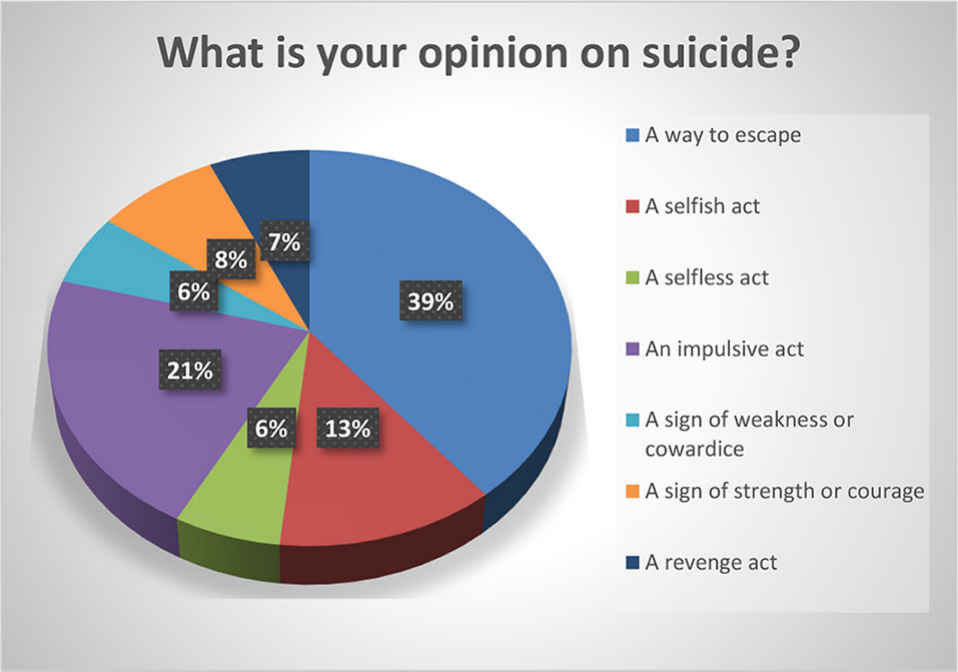
What is your opinion on suicide?

The respondents were predominantly white (59.33%) and in the age group 18–29 (63.09%). The majority had attended college: 23.84% had some college, 27.15% had a bachelor’s degree, and 25.17% had a graduate degree. 31.79% reported that someone close to them had died of suicide. Most (59.60%) did not believe that the media glorifies death by suicide and 52.03% of the respondents believed that social media is a good platform for people to ask for help. Of the respondents, 86% believed that suicide is preventable, and 72.85% believed that it a person’s right to die by suicide. Most of the responders (87%) viewed suicide as “an escape”, 47.6% as an impulsive act, 27.89% as a selfish act, 18.37% as a sign of strength, 14.97% as a revenge act, 13.61% as a selfless act, and 12.93% as a sign of weakness. Please see **Figures 1**–**5**.

Significant findings (p ≤ 0.05) included the following: those who identified as female found social media to be a good platform for people to ask for help (61%) while males did not (60.61%). Both whites (79.78%) and Asians (61.29%) believed it was a person’s right to die by suicide. Whites and Asians also viewed suicide to be a sign of weakness. Regarding education, those who had some college (83.33%) and those with a bachelor’s degree (78.05%) both believed that it was a person’s right to die by suicide. Interestingly, the opinion was split 50-50 among those with a graduate degree. Those who believed it was a person’s right to die to die by suicide viewed suicide as a sign of strength (23.36%) as opposed to those who did not (5%). Those who responded that someone close to them died by suicide believed that the media glorified suicide (56.25%) while those who did not lose someone close to suicide did not believe that (66.99%). Those who believed that media glorified suicide, also has a more stigmatizing view of suicide as a selfish act (41.67%) as opposed to those who did not (18.39%). Respondents who did not think suicide was preventable also viewed suicide as either a sign of strength (42.86%) or a revenge act (33.3%).

## Discussion

The study respondents were predominantly young and educated, and their view of suicide was overwhelmingly non-stigmatizing, with majority choosing responses such as “escape” (87%) or an impulsive act (47.6%). Overall, there were less of stigmatizing views of suicide, such as a selfish act (27.89%), revenge (13.61%), and a sign of weakness (12.93%); and less of glorifying, with only 14.97% viewing it as a sign of strength and 13.61% as a selfless act. The view of suicide as being an “escape” is somewhat neutral, a theory which can be ascribed to Shneidman (36) who described the “psychache” that one wants to escape from, and Baumeister (37). This approach does not blame the individual or others/society who supposedly failed the individual, rather the concept of “escape” is one that frees everyone to accept the inevitable. Survivors’ guilt can also compound and reinforce the stigma either imagined or accurately perceived by bereaved friends, family, and therapists (38). We did not find any correlation in our study that those who had lost someone close to suicide had greater stigmatizing attitudes. Those who did not think suicide was preventable viewed suicide as either a sign of strength or as a revenge act.

Our study found that 31.79% had lost someone close to them to suicide, and this is in keeping with the data that a third of Americans know someone who has died of suicide (39). We did not explore how the closeness impacted them. It has been shown that younger age at time of exposure to the suicide, time since the event, female sex, relationship with the deceased, and multiple exposures had greater personal impact (40). Those who had lost someone close to them to suicide believed that the media glorified suicide (56.25%) while those who did not lose someone close to suicide did not believe that (66.99%). Exposure to the suicide of a close friend or relative can influence attitudes to suicide which can impact own risk of suicide attempt; four key themes have emerged with a sense of gaining or losing control: 1) Suicide as a more tangible option; 2) Identification with the deceased and awareness of shared vulnerabilities to suicide; 3) Personal determination to avoid suicide; and 4) Beliefs regarding safeguards against suicide. Those who are determined to avoid suicide seek to exercise control over a perceived risk, aware of the devastating grief caused by suicide; others who perceived their own susceptibility to suicide described a sense of inevitability, which they either battled against or submitted to (38). Positive attitudes towards suicide, the “right to commit suicide” subscale can be predictive of suicide risk (41).

Of the respondents 40.4% believe that the media glorifies death by suicide. The “Werther Effect” or copycat phenomenon refers to the media reporting of suicides by celebrities and well-known figures which leads to an increase in suicide deaths in the general population (42). The exact mechanism of how celebrity suicides act to increase suicidal risk in the wider public is unknown, but emotional reactions may play a part (43). After the release of the Netflix series, “13 reasons why” about the aftermath of a 17-year-old’s suicide, there was an increase in suicide rate about 10–19-year-olds which appeared to be consistent with a contagion by media (44). It is important to train journalists in responsible professional media coverage: avoiding sensationalism and glorification, martyrification, and mystification of suicide; avoiding detailed descriptions of suicide methods used (45).

Women (61%) found social media to be a good platform for people to ask for help while only 39.39% of men did; on the other hand, 38.96% of the women did not find social media to be good platform to seek help, compared to 60.61% of men. Part of dispelling the stigma surrounding suicide is to get more people to talk about it. Our societies have perpetuated longstanding gender roles which are changing, however dominant masculine norms still exist which had led to men to avoid being emotional, and likely linked to lower likelihood of seeking help. The Australian “Man Up” Twitter campaign successfully influenced the social media conversation about masculinity and suicide, was game-changing in shifting attitudes toward expressing emotions and reaching out to others for help (46).

It is encouraging that 86% of the respondents believe suicide is preventable. We need to continue to raise awareness of it being a public health concern and to promote prevention efforts. A major intervention that needs ongoing support is restricting public access to lethal means of suicide: including firearm control legislation, restrictions on pesticides, detoxification of domestic gas, restrictions on prescription and sale of barbiturates, packaging analgesics in blister packets only and reducing number of tablets per package, mandatory use of catalytic converters in motor vehicles, construction of barriers at jumping sites (47).

More than half of the respondents (52%) feel that social media platforms are a good place to ask for help and seek support from others. Because of this, we need to harness technological advances such as machine learning to create safer spaces on social media platforms for those at risk for suicidality. Linguistic pattern recognition of stigma expressions around suicide attempts in Weibo (social media in China) posts have confirmed that social media mining can help improve stigma reduction programs (48). Machine learning is currently used to automatically identify and score helpful comments in a subreddit suicide watch forum to assist moderators with immediate feedback for help with online suicide prevention (49). Suicide happens because of numerous factors coming together, and it is hard to precisely pin down ways it can be prevented. Rather than to focus on risk factors, we need to shift emphasis on risk algorithms by employing machine learning algorithms to form complex, albeit robust and replicable combinations of many potential risk factors within large data sets (50).

Limitations of this study include the fact that those who respond to a survey voluntarily might have an interest in the topic, and this might not accurately reflect the public attitude. Because it is an anonymous online survey, there is no way to verify data. We did not perform further analysis of the data. In addition, we only surveyed those who are Facebook and Reddit users, and did not post on the numerous social media platforms available

## Conclusions

The social media is a medium with boundless possibilities, which melds together an individual’s biological and psychological uniqueness in the collective wisdom of a group that reflects the sociocultural mores of the time— we need to better understand the views held within such forums before we can tap in to bring about meaningful changes in how we approach public health concerns such as suicide.

We gauged the perception of suicide on social media platforms and found among the sample of mostly young white respondents, suicide is not as stigmatized, most believed it is preventable, while also supporting the right to die by suicide. While women found social media a good platform to ask for support, men did not, which reflects data that women tend to be more willing to seek help. A third of the group had lost someone close to them to suicide which was the national average and they tended to believe that media glorified suicide.

More research is needed to better understand how the social media influences its users, and whether it could be utilized as a forum to reduce stigma, promote open and nuanced discussions that promote wellness. Social media platforms need to use machine learning algorithms more for intervention purposes.

## Data Availability Statement

All datasets generated for this study are included in the article/supplementary material.

## Ethics Statement

The study involving human participants was reviewed and approved by Stanford University Institutional Review Board. The participants provided their written informed consent to participate in this study.

## Author Contributions

NN designed the study, implemented it and collected data. Both authors, NN and KN, collaborated on the data analysis and wrote the paper together.

## Conflict of Interest

The authors declare that the research was conducted in the absence of any commercial or financial relationships that could be construed as a potential conflict of interest.
